# Characterisation of the cannabinoid receptor system in synovial tissue and fluid in patients with osteoarthritis and rheumatoid arthritis

**DOI:** 10.1186/ar2401

**Published:** 2008-04-16

**Authors:** Denise Richardson, Richard G Pearson, Nisha Kurian, M Liaque Latif, Michael J Garle, David A Barrett, David A Kendall, Brigitte E Scammell, Alison J Reeve, Victoria Chapman

**Affiliations:** 1 Centre for Analytical Bioscience, School of Pharmacy, University of Nottingham, Nottingham, NG7 2RD, UK; 2 Division of Orthopaedic and Accident Surgery, School of Medical Surgical Sciences, University of Nottingham, Queen's Medical Centre Campus, Nottingham, NG7 2UH, UK; 3 School of Biomedical Sciences, University of Nottingham, Nottingham NG7 2UH, UK; 4 Neurology Centre of Excellence for Drug Discovery, GlaxoSmithKline Pharmaceuticals, 3rd Avenue, Harlow, Essex CM19 5AW, UK

## Abstract

**Introduction:**

Cannabis-based medicines have a number of therapeutic indications, including anti-inflammatory and analgesic effects. The endocannabinoid receptor system, including the cannabinoid receptor 1 (CB_1_) and receptor 2 (CB_2_) and the endocannabinoids, are implicated in a wide range of physiological and pathophysiological processes. Pre-clinical and clinical studies have demonstrated that cannabis-based drugs have therapeutic potential in inflammatory diseases, including rheumatoid arthritis (RA) and multiple sclerosis. The aim of this study was to determine whether the key elements of the endocannabinoid signalling system, which produces immunosuppression and analgesia, are expressed in the synovia of patients with osteoarthritis (OA) or RA.

**Methods:**

Thirty-two OA and 13 RA patients undergoing total knee arthroplasty were included in this study. Clinical staging was conducted from x-rays scored according to Kellgren-Lawrence and Larsen scales, and synovitis of synovial biopsies was graded. Endocannabinoid levels were quantified in synovial fluid by liquid chromatography-mass spectrometry. The expression of CB_1 _and CB_2 _protein and RNA in synovial biopsies was investigated. Functional activity of these receptors was determined with mitogen-activated protein kinase assays. To assess the impact of OA and RA on this receptor system, levels of endocannabinoids in the synovial fluid of patients and non-inflamed healthy volunteers were compared. The activity of fatty acid amide hydrolase (FAAH), the predominant catabolic endocannabinoid enzyme, was measured in synovium.

**Results:**

CB_1 _and CB_2 _protein and RNA were present in the synovia of OA and RA patients. Cannabinoid receptor stimulation of fibroblast-like cells from OA and RA patients produced a time-dependent phosphorylation of extracellular signal-regulated kinase (ERK)-1 and ERK-2 which was significantly blocked by the CB_1 _antagonist SR141716A. The endocannabinoids anandamide (AEA) and 2-arachidonyl glycerol (2-AG) were identified in the synovial fluid of OA and RA patients. However, neither AEA nor 2-AG was detected in synovial fluid from normal volunteers. FAAH was active in the synovia of OA and RA patients and was sensitive to inhibition by URB597 (3'-(aminocarbonyl) [1,1'-biphenyl]-3-yl)-cyclohexylcarbamate).

**Conclusion:**

Our data predict that the cannabinoid receptor system present in the synovium may be an important therapeutic target for the treatment of pain and inflammation associated with OA and RA.

## Introduction

Osteoarthritis (OA) is the most common form of arthritis affecting synovial joints [1]. The aetiology of OA is poorly understood, with mechanical, metabolic, and inflammatory causes. Inflammation and angiogenesis and their possible role in disease progression and pain are increasingly being recognised as important aetiological factors [2-5]. Rheumatoid arthritis (RA) is a systemic, autoimmune-mediated, inflammatory arthritis [6]. Although the pathogenesis remains incompletely understood, it is characterised by severe, progressive synovitis with rapid destruction of the joint. Pro-inflammatory cytokines such as tumour necrosis factor (TNF)-α, interleukin (IL)-1, IL-6, and chemokines such as IL-8 are abundant in RA tissue, which is compensated to some degree by the increased production of anti-inflammatory cytokines such as IL-10 and transforming growth factor-β [7]. The accepted therapeutic approach to RA is to use disease-modifying anti-rheumatic drugs at an early stage, and the recent introduction of cytokine inhibitor drugs has increased the effectiveness of treatment considerably. However, an effective remission-inducing drug has yet to be discovered, and the vast majority of RA patients are dependent on lifelong treatment in order to suppress joint damage and functional impairment [6]. There are no proven disease-modifying OA drugs, and current non-steroidal anti-inflammatory drug (NSAID) treatments do not always provide adequate pain relief and have detrimental side effects. Thus, there is a strong rationale for the development of novel drug treatments for arthritis. This can be achieved only by an improved mechanistic understanding of the functional cellular changes associated with this disease.

The cannabinoid receptor system has been implicated in a wide range of physiological and pathophysiological processes [8]. Recent pre-clinical and clinical studies have demonstrated that cannabis-based drugs have therapeutic potential in inflammatory diseases, including RA and multiple sclerosis [9]. Animal studies have demonstrated that activation of cannabinoid receptors attenuates inflammation and nociceptive processing in models of cutaneous and joint inflammation [10-14]. The cannabis-based medicine Sativex (GW Pharmaceuticals plc, Salisbury, Wiltshire, UK) has been reported to produce a significant analgesic effect and to suppress disease activity in patients with RA [15].

Two cannabinoid receptors (CB_1 _and CB_2_), both of which are inhibitory G protein-coupled receptors, have been cloned [8]. CB_1 _receptors are expressed predominantly by peripheral nerves, spinal cord, and the nervous system as well as peripheral immune cells [16]. CB_2 _receptors are expressed mainly in peripheral tissue, in particular by immune cells [17]. Activation of CB_1 _receptors is associated predominantly with a dampening down of neuronal excitability, whereas activation of CB_2 _receptors is associated with decreases in immune cell function, including attenuated cytokine release [9,17]. A number of endocannabinoids with activity at the CB_1 _and CB_2 _cannabinoid receptors, including *N*-arachidonyl ethanolamide (anandamide, AEA) and 2-arachidonyl glycerol (2-AG), have been identified [18,19]. Other structurally related endogenous fatty acid compounds such as oleoyl ethanolamide (OEA) and palmitoyl ethanolamide (PEA) have been identified in biological tissues. These compounds do not bind to cannabinoid receptors but might be involved in facilitating the actions of directly acting endocannabinoids and thus are commonly termed 'entourage' compounds due to their ability to modulate the endocannabinoid system [20,21]. The endocannabinoids and PEA are synthesised on demand, and AEA, PEA, and OEA are metabolised predominantly by fatty acid amide hydrolase (FAAH) [22,23].

Although the therapeutic benefits of Sativex in RA patients are significant, the mechanisms mediating these effects are unclear. Indeed, the impact of arthritis on the endocannabinoid receptor system, both in terms of receptor expression and levels of endocannabinoids and entourage compounds, is unknown. The endocannabinoid system appears to regulate bone mass by signalling via peripheral CB_2 _receptors in both osteoblasts and osteoclasts [24]. In a separate study, CB_1 _knockout mice had significantly increased bone mineral density compared with wild-type mice and were protected from ovariectomy-induced bone loss and CB_1_- and CB_2_-selective cannabinoid receptor antagonists inhibited osteoclastogenesis *in vivo *[25]. Thus, the role of the cannabinoid receptor system in bone remodelling and aspects of pathological conditions such as periarticular bony erosions in RA and subchondral bony sclerosis in OA remains unclear.

Several NSAIDs, including ibuprofen, ketorolac, indometacin, and niflumic acid, which act via the inhibition of cyclooxygenase (COX), have been shown to inhibit FAAH [26,27]. This suggests that current treatment of inflammatory pain in OA and RA patients using NSAIDs may be targeting endocannabinoid metabolism in addition to arachidonic acid metabolism. These interactions may be of great clinical importance in terms of multiple-target drug development as synergistic actions of the COX-2 inhibitor rofecoxib and the endocannabinoid AEA have been observed in an animal model of pain [28].

The aim of the present study was to provide evidence of a role for the cannabinoid receptor system in OA and RA. Here, we report the presence of an active endocannabinoid system, including endocannabinoids, entourage compounds, CB_1 _and CB_2 _receptors, and FAAH, in the knee synovia of patients with end-stage OA and RA.

## Materials and methods

### Patient information and tissue collection

The Nottingham Regional Ethical Committee approved the study, and after informed consent synovial biopsies and fluid were sampled from patients undergoing total knee arthroplasty (TKA). All x-rays were scored according to Kellgren and Lawrence [29] (OA) and Larsen [30] (RA) scales.

The synovial fluid and biopsies were collected under tourniquet control at the onset of the TKA from 32 OA and 13 RA patients. The synovial fluid samples were centrifuged at 1,000 *g *for 40 minutes at 4°C, and the supernatants were retained for analysis. Samples of synovial fluid from non-inflamed normal volunteers (n = 6) were kindly provided by Michael Doherty, Academic Rheumatology, Nottingham University Hospitals.

### Synovium histology and analysis

**Figure 1:**
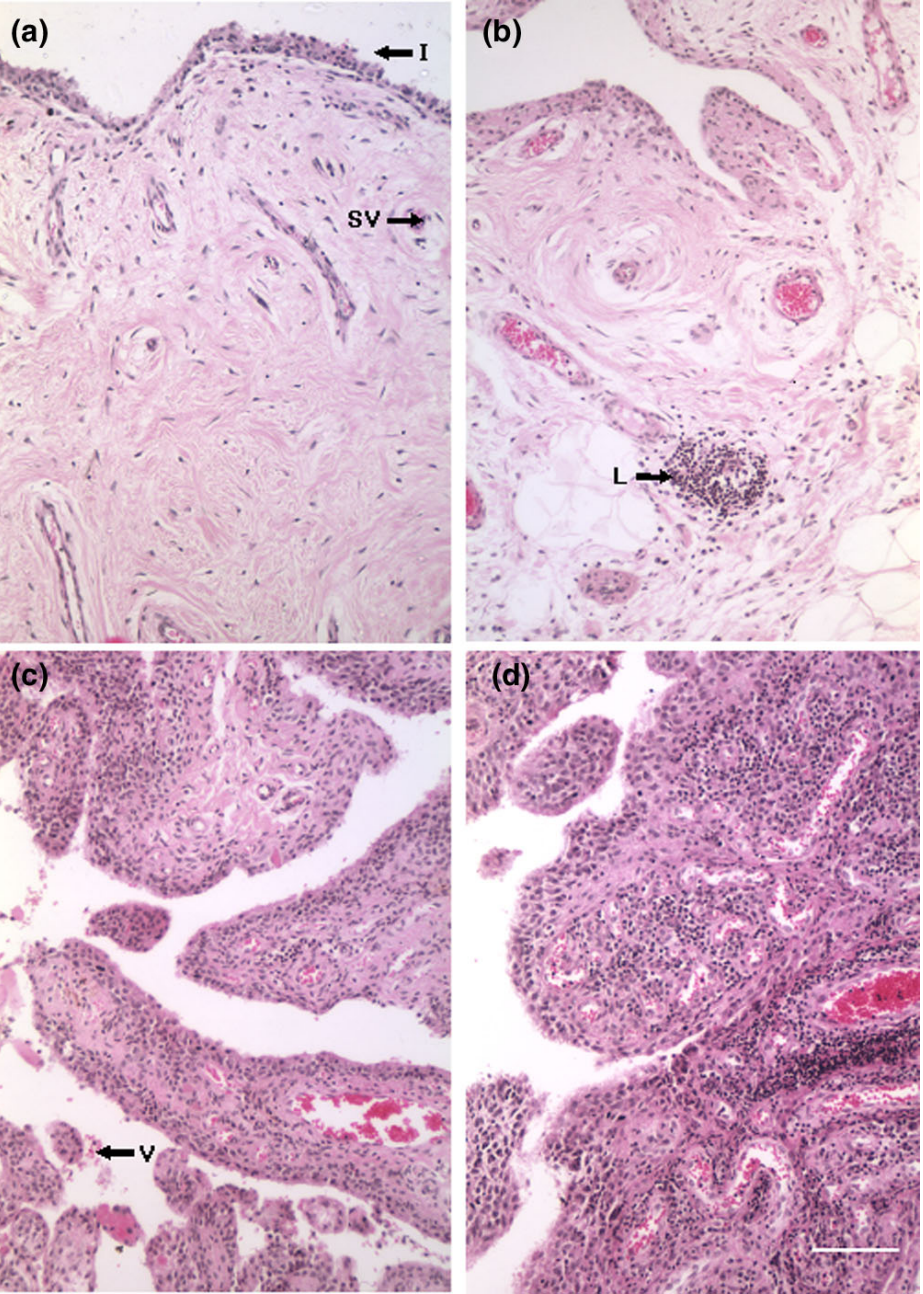
Representative haematoxylin and eosin micrographs of synovium biopsies taken at osteoarthritis or rheumatoid arthritis total knee arthroplasty. **(a-c) **Osteoarthritis: **(a) **category 1 (least inflamed), **(b) **category 2, and **(c) **category 3 (most inflamed). I, synovium intima; L, lymphoid body; SV, small vessel; V, villus. **(d) **Rheumatoid arthritis, category 3. Each biopsy was assigned to one of four categories: 0 = normal: synovial intima less than four cells thick, sparse cellular distribution, with few or no inflammatory cells (not shown as normal synovial fluid samples were not accompanied by synovium biopsies); 1 = mild inflammation: synovial intima three to five cells thick, slight increase in cellularity with few inflammatory cells; 2 = moderate inflammation: synovial intima four to six cells thick, dense cellularity with inflammatory cells, may exhibit as small lymphoid aggregates; 3 = severe inflammation: synovial intima five to seven or more cells thick, dense cellularity with inflammatory cells, containing many or large perivascular lymphoid aggregates. Bar = 100 μm.

Synovial biopsies designated for histological analysis were fixed in 10% formal saline (Sigma-Aldrich, St. Louis, MO, USA), processed into paraffin wax, and stained with Weigert's haematoxylin and eosin. Each biopsy was assigned to one of four categories: 0 = normal: synovial intima less than four cells thick with sparse cellular distribution and few or no inflammatory cells; 1 = mild inflammation: synovial intima three to five cells thick with slight increase in cellularity and with few inflammatory cells; 2 = moderate inflammation: synovial intima four to six cells thick, with dense cellularity and inflammatory cells, may exhibit as small lymphoid aggregates; and 3 = severe inflammation: synovial intima five to seven or more cells thick, dense cellularity with inflammatory cells, containing many or large perivascular lymphoid aggregates [3]. Representative micrographs of this grading system are presented in Figure 1.

### Quantification of inflammatory cytokines in synovial fluid

Cytokine profiles in synovial fluid were determined using a BD™ cytometric bead array (BD Biosciences, San Jose, CA, USA), which quantified IL-8, IL-1β, IL-6, IL-10, TNF, and IL-12p70. Analysis was performed using a Beckman Coulter *Epics*™ Altra^® ^flow cytometer (Beckman Coulter, Fullerton, CA, USA) according to the manufacturer's protocol for measurements in serum or plasma.

### Measurement of endocannabinoids

A lipid extraction method was used as previously described [31]. In brief, tissue or fluid was homogenised in an ethyl acetate/hexane mixture with internal standards (0.42 nmol AEA-d8, 1.5 nmol 2-AG-d8, and 0.2 nmol heptadecanoyl ethanolamide) and left in extraction solvent for 2 hours with intermittent mixing. Repeated centrifugation and supernatant collection were then undertaken, followed by purification of samples by solid-phase extraction.

Simultaneous measurement of AEA, 2-AG, OEA, and PEA was then performed using liquid chromatography-tandem mass spectrometry. A triple quadrupole Quattro Ultima mass spectrometer (Waters Ltd, Manchester, UK) was used in electrospray-positive mode and coupled to an Agilent 1100 LC system (Agilent Technologies, Böblingen, Germany) for analysis. Analytes were chromatographically separated on a HyPurity Advance C8 column (Fisher Scientific UK., Loughborough, UK) with gradient elution. Individual compounds were then identified and quantified with multiple reaction monitoring, using (*m/z *[mass-to-charge] ratios of specific precursor and product ions) on the mass spectrometer.

### Western blotting for measurement of cannabinoid receptor expression

Human synovium samples were homogenised in lysis buffer (20 mM Tris, 1 mM ethylene glycol tetraacetic acid [EGTA], 320 mM sucrose, 0.1% Triton X100, 1 mM sodium fluoride, and 10 mM β-glycerophosphate) containing a protease inhibitor cocktail (Roche Applied Sciences, Burgess Hill, UK). Homogenates were centrifuged at 5,000 *g *for 10 minutes at 4°C and the resulting supernatants were collected. Estimation of protein content was carried out using the Lowry method [32]. Aliquots of the homogenate supernatant were diluted in Laemmli sample buffer, and proteins were separated using 10% SDS-PAGE and blotted onto nitrocellulose membranes. Anti-cannabinoid receptor 1 (Calbiochem, now part of EMD Biosciences, Inc., San Diego, CA, USA), anti-cannabinoid receptor 2 (Cayman Chemical Company, Ann Arbor, MI, USA), or anti-β-actin antibody (Sigma-Aldrich) was incubated overnight at 4°C with nitrocellulose membranes and visualisation using horseradish peroxidase-conjugated secondary antibodies (1:2,000 dilution; Dako Denmark A/S, Glostrup, Denmark), enhanced chemiluminescence detection (Amersham Biosciences, now part of GE Healthcare, Little Chalfont, Buckinghamshire, UK), and autoradiography. Data were quantified using a Bio-Rad GS 710 imaging densitometer (Bio-Rad Laboratories, Inc., Hercules, CA, USA).

### Fatty acid amide hydrolase activity assay

Tissues were homogenised (50 mM Tris-HCl, 1 mM EDTA [ethylenediaminetetraacetic acid], 3 mM MgCl_2_, pH 7.4) and centrifuged at 500 *g *for 5 minutes at 4°C, and the supernatant was subsequently centrifuged for 30 minutes at 35,000 *g *at 4°C. The pellet obtained was re-suspended in Tris-HCl buffer, and protein content was determined by the method of Lowry.

The FAAH activity of each sample was measured by monitoring the release of [^3^H]-ethanolamine after incubation of homogenate with radiolabelled AEA ([^3^H]-AEA). Protein contents per assay were chosen on the basis of preliminary experiments using some of the samples to establish optimal conditions (~88 μg/mL). Homogenised tissue in assay buffer (116 mM NaCl, 5.4 mM KCl, 1.8 mM CaCl_2_, 25 mM HEPES, 1 mM NaH_2_PO_4_, 0.8 mM MgSO_4_, pH 7.0) was incubated at 37°C with 40 μM [^3^H]-AEA (American Radiolabeled Chemicals, Inc., St. Louis, MO, USA) in the presence of 1 mg/mL fatty acid-free bovine serum albumin (200 μL total assay volume), and the reaction was stopped by the addition of 0.4 mL activated charcoal (4% wt/vol in 0.5 M HCl) [33,34]. A sample without homogenate was processed to determine the extent of non-enzymatic [^3^H]-AEA hydrolysis. The samples were vortexed and left to sediment for 30 minutes before centrifugation at 11,000 *g *for 5 minutes to pellet the charcoal and homogenate protein with un-hydrolysed [^3^H]-AEA. A 200-μL aliquot of the supernatant was counted for tritium content by liquid scintillation spectroscopy. For obtaining standards, an aliquot of the homogenate was incubated without [^3^H]-AEA and stopped with charcoal as for other samples. After centrifugation, 190 μL of supernatant was added into scintillation vials with 40 μM [^3^H]-AEA (equivalent to 2 nmol [^3^H]-AEA/mL) and activity was determined as before.

### Preparation and culture of human synovial fibroblast cells

Human synovial samples from both OA and RA patients were chopped and finely digested for 2 hours at 37°C with 2 mg/mL collagenase type H (Sigma-Aldrich) in Dulbecco's modified Eagle's medium (Sigma-Aldrich) supplemented with 10% foetal calf serum (PAA Laboratories Ltd, Yeovil, UK, Pasching, Austria), 2 mM L-glutamine (Sigma-Aldrich), 50 U/mL penicillin, and 50 μg/mL streptomycin and fungizone (Sigma-Aldrich). Samples were occasionally agitated to aid digestion. At the end of the digest, the samples were pipetted up and down to disrupt the tissue and passed through a 100-μm cell strainer (BD Falcon, a trademark of BD Biosciences). The cell suspension was centrifuged at 500 *g *for 5 minutes at room temperature, and the pellet was re-suspended in complete media, plated into flasks, and allowed to become adherent. Media was replaced the following day to remove any non-adherent cells. Adherent cells were cultured and used between passages 3 and 12.

### Immunoblotting of synovial fibroblast for mitogen-activated protein kinase activation

To analyse mitogen-activated protein kinase (MAPK) activation, synovial fibroblast-like cells were stimulated with the CB_1_/CB_2 _receptor agonist HU210 (1 μM) in the presence and absence of a 20-hour pre-incubation with pertussis toxin (PTX) for 5, 10, 20, and 40 minutes before analysis of MAPK phosphorylation (described below) to determine a maximum time-dependent effect of HU210 stimulation on MAPK phosphorylation compared with basal, unstimulated levels. In subsequent experiments, synovial fibroblast-like cells were stimulated with HU210 (0.1 μM) in the presence and absence of the CB_1 _antagonist SR141716A (1 μM) or CB_2 _antagonist SR144528 (1 μM). Cells were washed with phosphate-buffered saline (Sigma-Aldrich) and lysed (20 mM Tris, 1 mM EGTA, 320 m sucrose, 0.1% Triton X100, 1 mM sodium fluoride, and 80 mM β-glycerophosphate containing protease inhibitor cocktail). After removal of a sample for a protein assay, the homogenate was diluted in Laemmli sample buffer and heated at 95°C for 5 minutes. Equal amounts of protein from each sample were separated on 10% SDS-PAGE gels and then transferred onto nitrocellulose membranes for Western blotting. Nitrocellulose blots were incubated overnight at 4°C with an antibody that recognises the double-phosphorylated (activated) forms of both isoforms of extracellular signal-regulated kinase (ERK) (p44 ERK1 MAPK and p42 ERK2 MAPK) and p38 MAPK (New England Biolabs, Inc., Ipswich, MA, USA). Proteins were subsequently visualised using the ECL system (Amersham Life Sciences, now part of GE Healthcare). Blots were then stripped of antibodies using Restore Western Blot Stripping Buffer (Pierce, Rockford, IL, USA) according to the manufacturer's instructions. These blots were subsequently re-probed with an antibody against total ERK and p38 (New England Biolabs, Inc.). Bands were visualised as before. Data were quantified using the Bio-Rad GS 710 imaging densitometer and represented as a percentage of the unstimulated control.

### Reverse transcription-polymerase chain reaction for CB_1 _and CB_2 _receptors

Total RNA was isolated from cultured human synovial-like fibroblasts using TRiPure Isolation reagent (Roche Applied Sciences, Burgess Hill, UK) according to the manufacturer's instructions. As the open reading frame for CB_1 _and CB_2 _cannabinoid receptors for humans contains a single exon, the RNA used was treated with recombinant RNase-free DNase-1 (Roche) to remove any genomic DNA contamination and was purified using a standard phenol-chloroform extraction methodology. RNA was reverse-transcribed into cDNA using the Transcriptor first-strand cDNA synthesis kit (Roche Applied Sciences, Burgess Hill, UK) according to the manufacturer's instructions. Amplification of CB_1 _and CB_2 _cannabinoid receptor cDNA was achieved by using 'touchdown' polymerase chain reaction (PCR) with a progressive decrease in annealing temperatures from 60°C until touchdown at 55°C [35]. Primers were designed using the Primer Express software package version 3.0 (Applied Biosystems, Foster City, CA, USA). Using bioinformatics, the specificities of all primers were confirmed to the desired mRNA of human samples. Primers used were as follows: CB_1 _forward: 5'-CTGAACTCCACCGTGAACC-3' and reverse: 5'-GTGCTCTTGATGCAGCT-5' and CB_2 _forward: 5'-CATCTATGCTCTACGGAGTGGAGAG-3' and reverse: 5'-TTGAGTTGTTTAAATTGGGAAGAGG-3' (MWG-Biotech AG, Ebersberg, Germany). The amplified products were separated on a 1.8% low-melting agarose gel (Invitrogen Corporation, Carlsbad, CA, USA) stained with ethidium bromide and documented using GeneSnap imaging software (Syngene, Cambridge, UK).

### Data analysis

Statistical comparisons of levels of cytokines between normal, OA, and RA samples were performed with a non-parametric Kruskal-Wallis test. Comparisons of endocannabinoid (EC) levels between normal, OA, and RA samples were performed with a non-parametric Mann-Whitney test. Comparison of FAAH activity between OA and RA synovial tissue was performed using unpaired Student *t *tests. Comparisons between drug treatment groups in cultured fibroblast-like cell immunoblots were performed using one-way analysis of variance followed by Bonferroni multiple comparison *post hoc *test. A *P *value of less than 0.05 was considered a significant difference.

## Results

### Patient information

All patients had a Kellgren-Lawrence or Larsen radiological score of greater than or equal to 3. The study included 14 male and 18 female OA patients and 1 male and 12 female RA patients. The patients had a similar mean age, and information on drug history prior to TKA is summarised in Table 1. Patients were told to stop taking aspirin and all cytokine inhibitors 10 to 14 days prior to surgery.

**Table 1:** Summary of patient histories of osteoarthritis and rheumatoid arthritis patients included in the study

	Osteoarthritis		Rheumatoid arthritis	
Gender (number)	Female (18)	Male (14)	Female (12)	Male (1)
Mean age ± standard deviation, years	72 ± 7	70 ± 8	61 ± 11	64
NSAIDs, number (percentage)	8 (44%)	7 (50%)	8 (67%)	0
Paracetamol, number (percentage)	9 (50%)	7 (50%)	9 (75%)	1 (100%)
Corticosteroid, number (percentage)	0	0	8 (67%)	0
NSAIDs, paracetamol or corticosteroid, number (percentage)	12 (67%)	9 (64%)	12 (100%)	1 (100%)
Anti-cytokine therapy, number (percentage)	0	0	1 (8%)	1 (100%)

NSAID, non-steroidal anti-inflammatory drug.

### Histology of synovial biopsies and levels of cytokines

Haematoxylin and eosin histology was performed on 26 of the OA and 9 of the RA synovial biopsies to assess the degree of inflammation (Figure 1). None of the OA synovia was characterised as having a normal histological appearance, 4 exhibited mild (score = 1), 12 moderate (score = 2), and 10 severe (score = 3) synovial inflammation. In the case of the RA synovial biopsies, one sample had become fibrotic and devoid of a cellular component and therefore could not be scored. Six of the RA biopsies were described as exhibiting severe synovitis (score = 3) and two as having moderate inflammation (score = 2).

**Figure 2:**
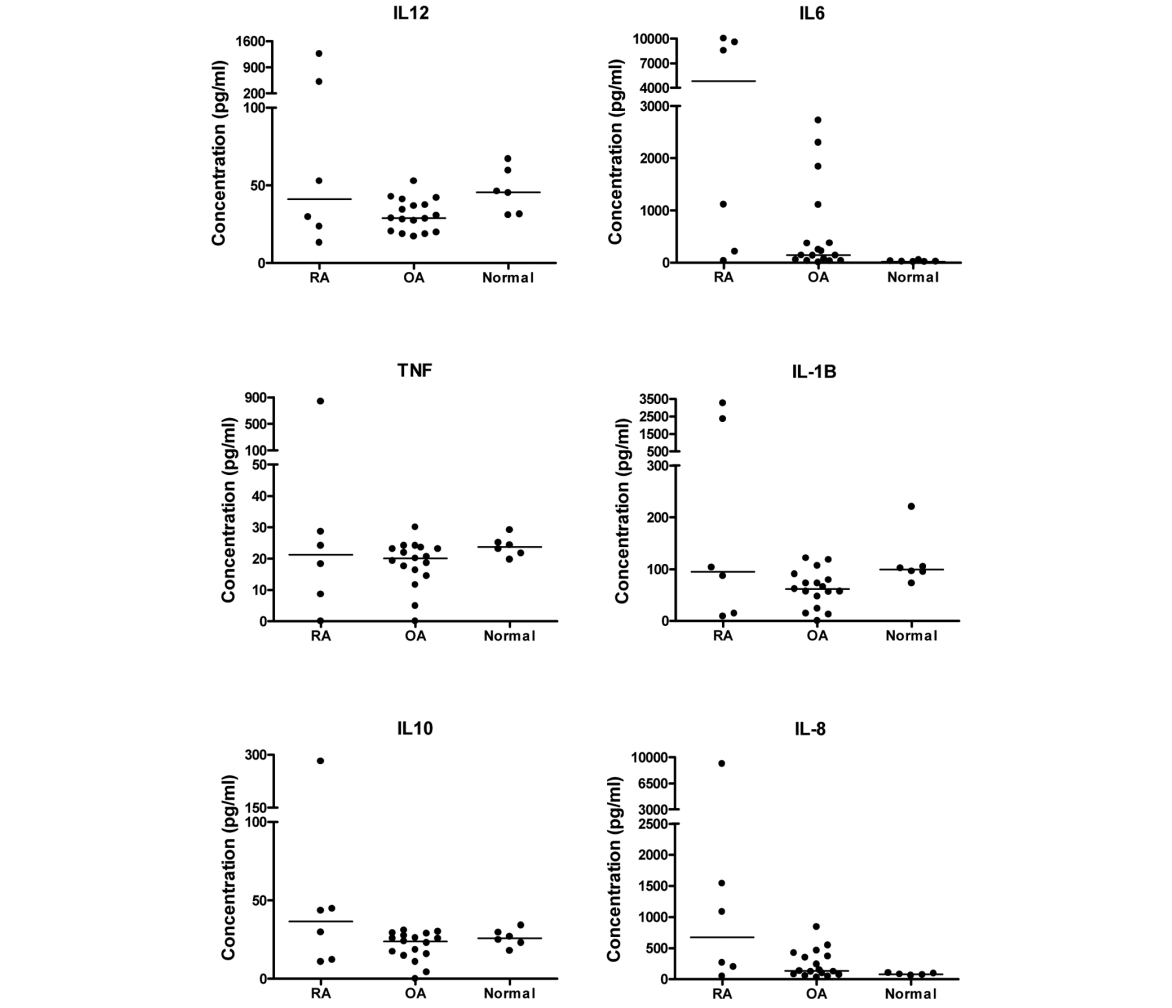
Comparison of levels of cytokines in synovial fluid from patients with rheumatoid arthritis (RA) (n = 6) and osteoarthritis (OA) (n = 17) and from non-inflamed normal volunteers (normal) (n = 6). Levels of interleukin (IL)-6 were significantly higher in RA and OA groups (*P *< 0.05 for both) compared with the normal group (Kruskal-Wallis non-parametric test and Dunn multiple comparison *post hoc *test). Lines represent median values. TNF, tumour necrosis factor.

In addition to assessing the degree of degeneration of the synovium of the patients included in this study, levels of inflammatory cytokines in the synovial fluid of these patients were quantified. Levels of cytokines (IL-8, IL-1β, IL-6, IL-10, TNF, and IL-12) were assayed in 17 of the OA synovial fluid samples, 6 RA samples, and 6 samples from patients with no clinical symptoms (described as normal). In general, levels of cytokines were higher in the synovial samples from RA patients compared with OA and normal samples (Figure 2) and there was a large spread of data in the RA group. Statistical comparison between levels of cytokines in the three groups revealed that levels of IL-6 were significantly higher in the RA (*P *< 0.01) and OA (*P *< 0.05) samples compared with normal synovial fluid samples (Figure 2). There were no significant differences between levels of the other cytokines for the three groups.

### Cannabinoid receptors are expressed in human synovial tissue from osteoarthritis and rheumatoid arthritis patients

**Figure 3:**
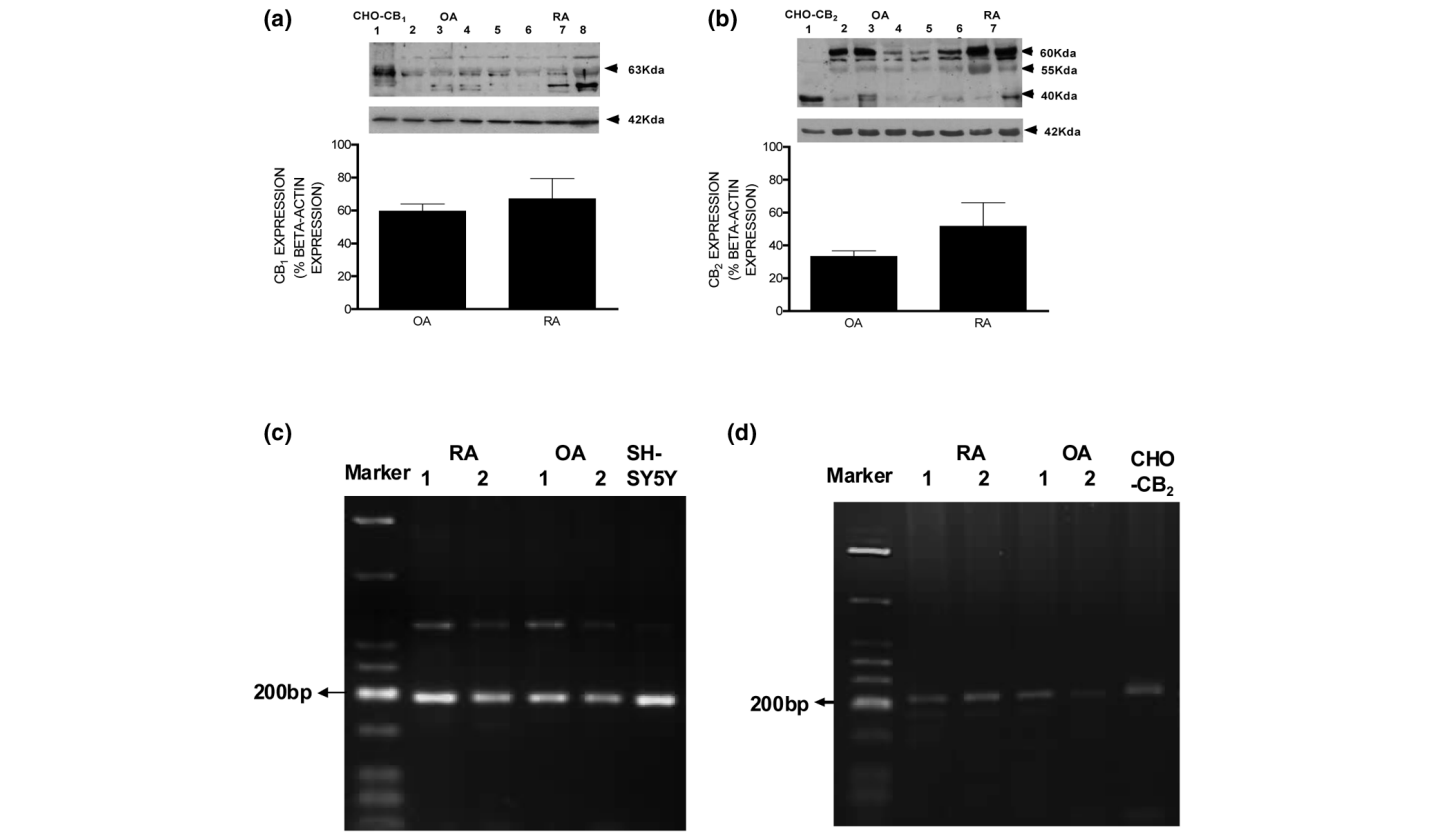
Expression of cannabinoid receptor 1 (CB_1_) **(a) **and cannabinoid receptor 2 (CB_2_) **(b) **receptor protein in synovial tissue of patients with osteoarthritis (OA) (lanes 2 to 5) or rheumatoid arthritis (RA) (lanes 6 to 8) detected by Western blotting and quantified by densitometry. CHO-K1 cells recombinantly expressing either CB_1 _or CB_2 _receptor were used as a positive control (lane 1). Receptor expression was calculated as a percentage of expression of the internal control, β-actin. Data are presented as mean ± standard error of the mean of the expression of CB_1 _and CB_2 _protein in four OA and four RA patients, assessed in three separate experiments. Expression of CB_1 _**(c) **and CB_2 _**(d) **receptor RNA in fibroblast-like synovial cells derived from tissue of patients with OA or RA detected by 'touchdown' polymerase chain reaction. A human neuroblastoma cell line (SH-SY5Y) endogenously expressing CB_1 _cannabinoid receptor and CHO-K1 cells recombinantly expressing CB_2 _receptor were used as positive controls, respectively. bp, base pairs.

The expression of cannabinoid receptors in human synovial tissue obtained from OA and RA patients was assessed. Immunoblotting for CB_1 _receptor protein in human synovial tissue detected a major band at about 63 kDa, consistent with previous reports (Figure 3a) [36-39]. The expression of CB_2 _receptor protein was also detected in synovial tissue by Western blotting. Three bands probably representing different glycosolation states, at about 40, 55, and 60 kDa (Figure 3a), were detected in a pattern similar to that previously reported for spleen, brainstem, and cerebellum [40]. Processing immunoblots without primary antibody or pre-absorbing with antigenic peptide abolished the identified bands (data not shown). CHO-K1 cells recombinantly expressing either the human CB_2 _receptor or human CB_1 _receptor protein were used as a positive control. There were no significant differences between levels of expression of CB_1 _and CB_2 _receptor proteins in synovial tissue from OA (n = 4) and RA (n = 4) patients. To further strengthen the evidence for CB_1 _and CB_2 _receptor expression in synovial tissue from OA and RA patients, touchdown PCR was used to detect RNA for CB_1 _and CB_2 _receptors. CB_1 _and CB_2 _RNA was observed in all human synovial fibroblast-like synovial cells analysed with a product size of 201 base pairs, as predicted (Figure 3b). The human neuroblastoma cell line SHSY-5Y, which endogenously expresses CB_1 _cannabinoid receptors [41], and CHO-K1 cells recombinantly expressing human CB_2 _cannabinoid receptors were used as positive controls. The lack of amplification in non-template controls and in the absence of reverse transcriptase indicates the absence of any contamination or amplification of genomic DNA (data not shown).

### Determination of fatty acid amide hydrolase activity in human synovial tissue

Membrane fragments prepared from synovial tissue were assayed for determining FAAH activity. A rat liver membrane preparation, previously demonstrated to be rich in FAAH activity, was used as a positive control [42]. The selective FAAH inhibitor URB597 (3'-(aminocarbonyl) [1,1'-biphenyl]-3-yl)-cyclohexylcarbamate) (1 μM) virtually abolished activity in this tissue (Table 2). Although FAAH activity was much lower in synovium, activity was measurable in tissue from OA and RA patients (Table 2). There were no significant differences in FAAH activity between synovial tissue from OA and RA patients. Incubation of samples with URB597 (1 μM) also markedly reduced FAAH activity (Table 2) in the synovium [43].

**Table 2:** Fatty acid amide hydrolase (FAAH) activity in synovial tissue from osteoarthritis (n = 5) and rheumatoid arthritis (n = 4) patients

	FAAH activity (pmol/minute/mg protein)	
Tissue	Control	+ URB597
Osteoarthritis synovium	2.8 ± 0.46	Undetectable
Rheumatoid arthritis synovium	1.6 ± 0.42	Undetectable
Rat liver	988 ± 84	1.28 ± 0.51

FAAH activity was measured by monitoring the release of [^3^H]-ethanolamine after incubation of homogenates with [^3^H]-anandamide. Each sample was processed in three separate experiments. Data are presented as mean ± standard error of the mean. URB597, 3'-(aminocarbonyl) [1,1'-biphenyl]-3-yl)-cyclohexylcarbamate.

### Endocannabinoid levels in synovium tissue and synovial fluid in normal, osteoarthritis, and rheumatoid arthritis samples

**Figure 4:**
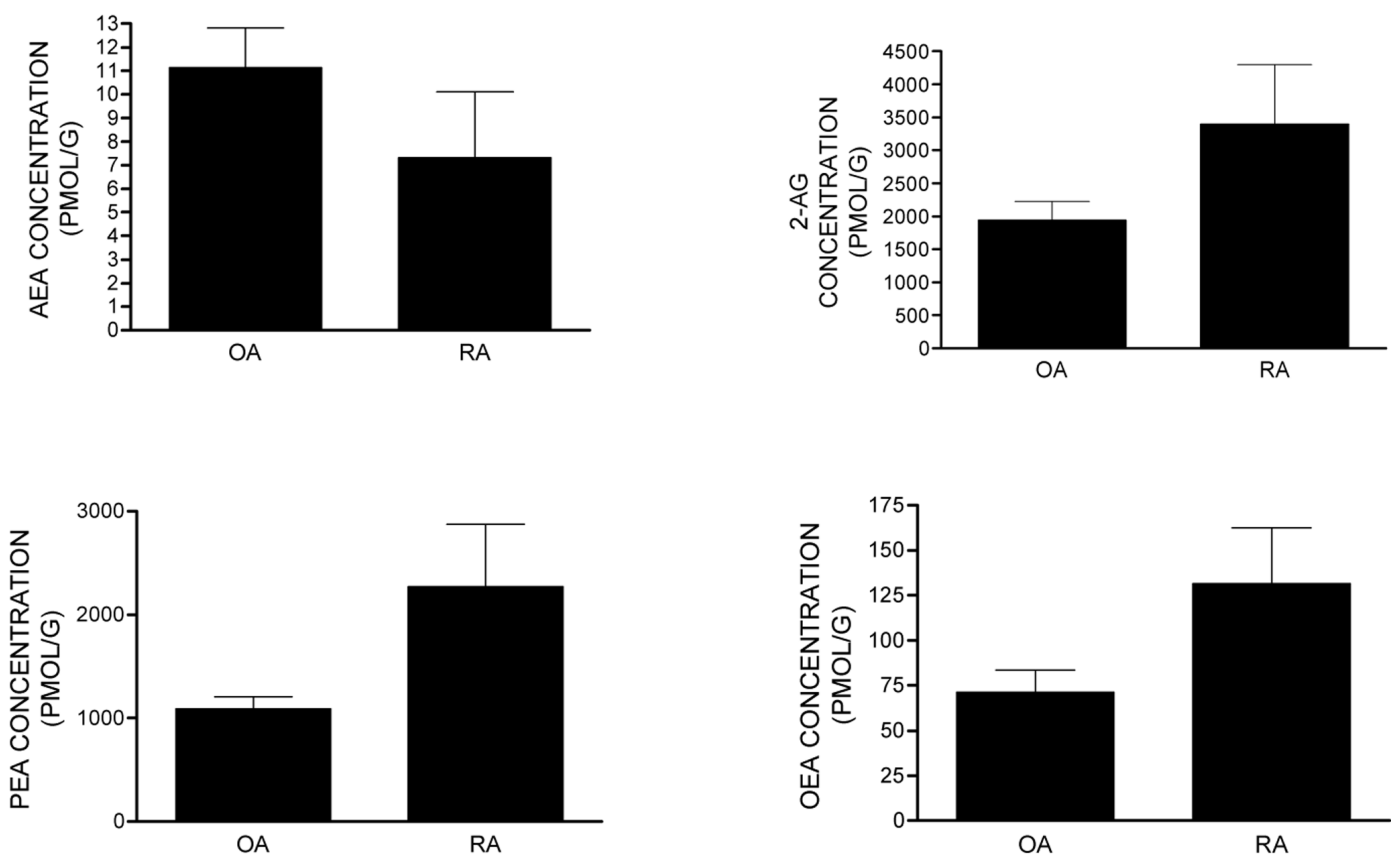
Levels of the endocannabinoids anandamide (AEA), 2-arachidonyl glycerol (2-AG), palmitoyl ethanolamide (PEA), and oleoyl ethanolamide (OEA) in the synovia of patients with either osteoarthritis (OA) (n = 14) or rheumatoid arthritis (RA) (n = 6). Data were analysed using the non-parametric Mann-Whitney test and are expressed as picomoles per gram.

The synovial tissue from OA and RA patients was used to measure endocannabinoid and entourage compounds. AEA, 2-AG, OEA, and PEA were detected and quantified in all samples analysed (Figure 4). Comparison of OA and RA tissue showed no significant differences in levels of AEA, 2-AG, OEA, or PEA.

**Figure 5:**
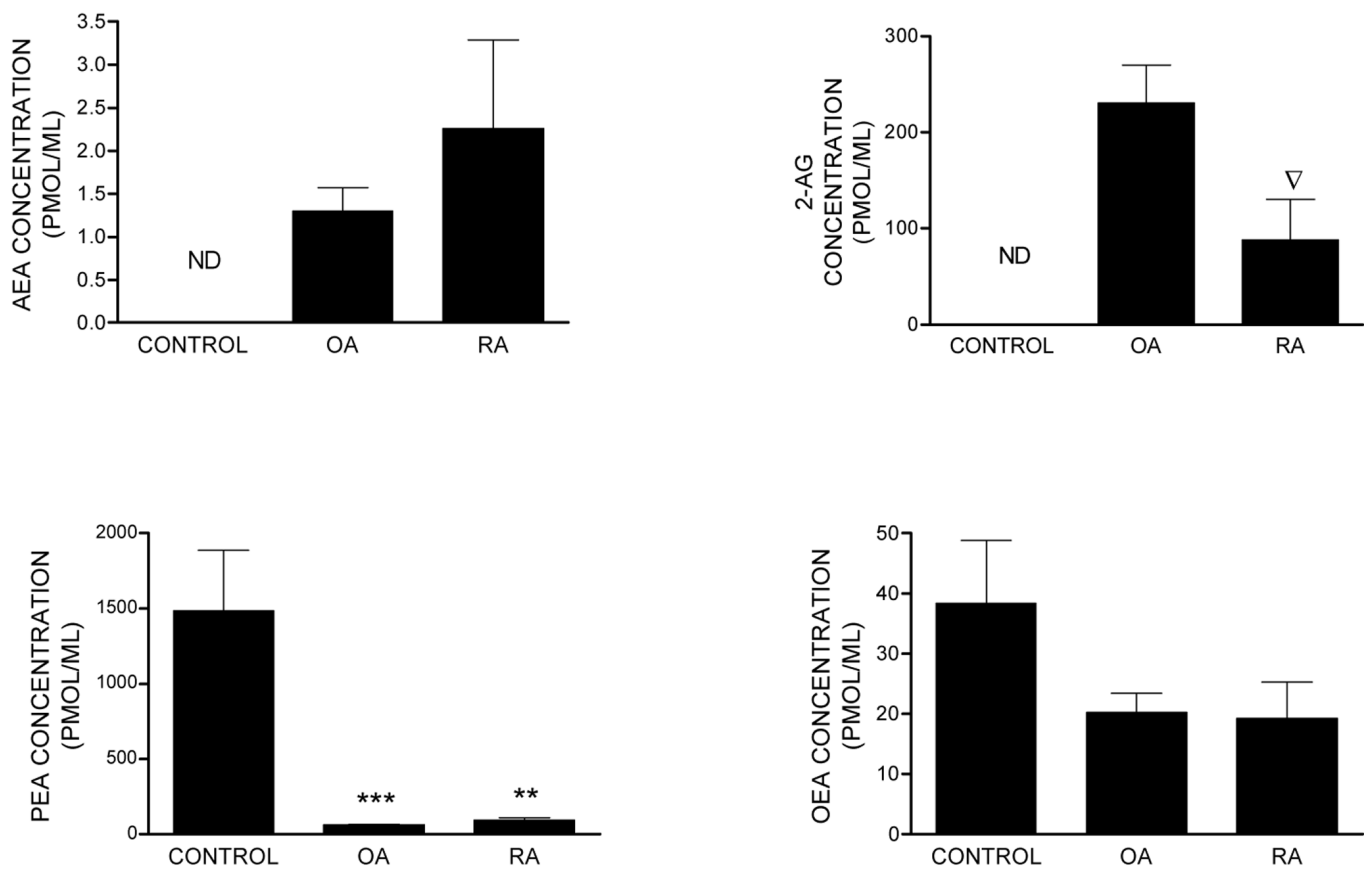
Levels of the endocannabinoids anandamide (AEA), 2-arachidonyl glycerol (2-AG), palmitoyl ethanolamide (PEA), and oleoyl ethanolamide (OEA) in synovial fluid of non-inflamed normal volunteers (control) (n = 6) and of patients with osteoarthritis (OA) (n = 14) and rheumatoid arthritis (RA) (n = 5). Data were analysed using the non-parametric Mann-Whitney test and are expressed as picomoles per millilitre. **P < 0.01, ***P < 0.005 compared with control values. ^▽ ^*P *< 0.05 compared with OA values. ND, not detected below a limit of detection of 0.3 pmol/mL.

Endocannabinoids and entourage compounds were measured in control synovial fluid from normal volunteers with no joint symptoms as well as in synovial fluid from OA and RA patients (Figure 5). AEA and 2-AG were not detected in the normal synovial fluid samples (below the lower limit of quantification of 0.3 pmol/mL). By contrast, significant levels of OEA and high levels of PEA were detected in these normal samples (Figure 5). Consistent with synovial tissue, AEA, 2-AG, OEA, and PEA were detected in synovial fluid samples taken from the same OA and RA patients (Figure 5). In contrast to the high levels of PEA in synovial fluid samples of normal volunteers, levels were greatly reduced in OA and RA samples. In addition, there was a trend toward a reduction in levels of OEA in OA and RA samples compared with control synovial fluid samples, although this did not reach statistical significance. Comparison of levels of endocannabinoid and entourage compounds in the synovial fluid versus synovia of OA and RA patients revealed that, generally, levels were lower (per millilitre of fluid) in the fluid compared with the synovial tissue (per gram of tissue).

### Effects of HU-210 on ERK1, ERK2, and p38 MAPK activation in fibroblast-like cells

**Figure 6:**
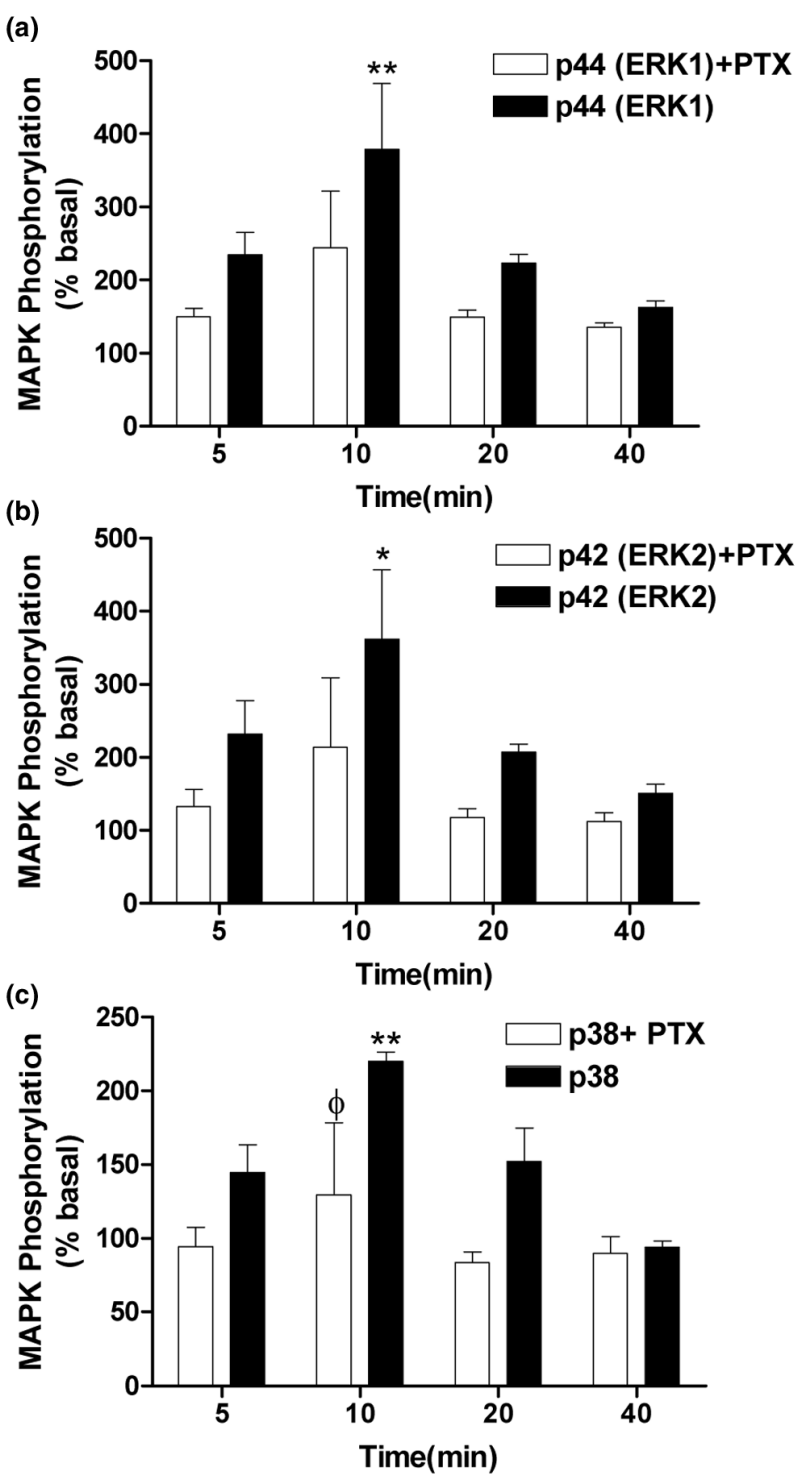
HU210 (1 μM) induces a time-dependent increase in **(a) **p44 (ERK1), **(b) **p42 (ERK2), and **(c) **p38 mitogen-activated protein kinase (MAPK) phosphorylation (filled bars) in fibroblast-like cells derived from the synovia of patients with rheumatoid arthritis or osteoarthritis compared with basal levels. Pre-incubation (20 hours) of cells with pertussis toxin (PTX) attenuated HU210-induced phosphorylation. Data are presented as mean percentage of unstimulated basal MAPK phosphorylation levels ± standard error of the mean (n = 3 synovia). Comparisons between groups were made using one-way analysis of variance. *P < 0.05, **P < 0.01 versus basal levels, ^φ ^*P *< 0.05 versus corresponding PTX-stimulated time point. ERK, extracellular signal-regulated kinase.

**Figure 7:**
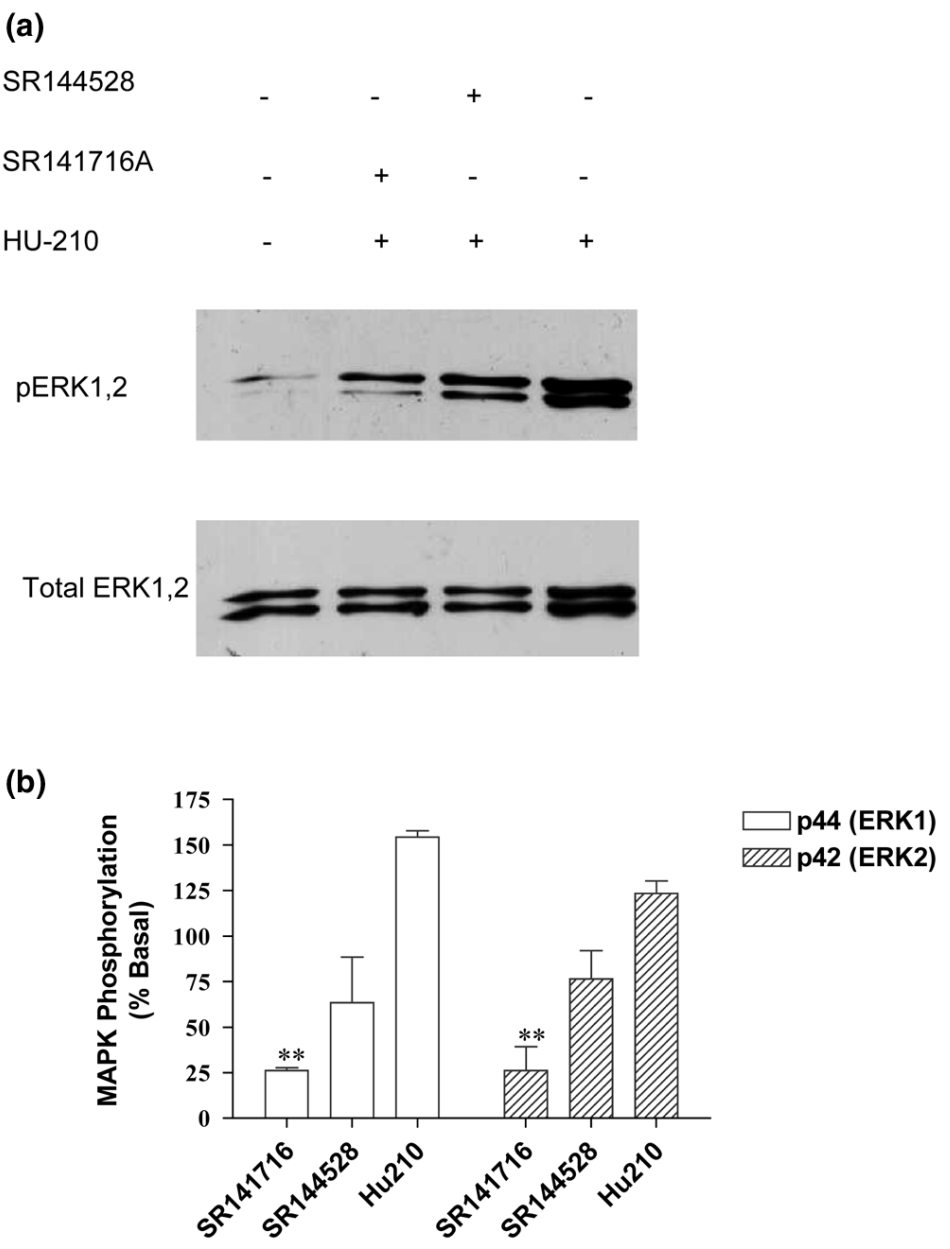
Pre-incubation of fibroblast-like cells with the cannabinoid receptor 1 (CB_1_) antagonist SR141716A (1 μM) for 10 minutes prior to exposure to HU210 (for a further 10 minutes, 0.1 μM) significantly blocked HU210-induced phosphorylation of p44 (ERK1) and p42 (ERK2). The cannabinoid receptor 2 (CB_2_) antagonist SR144528 (1 μM) did not significantly alter HU210-induced phosphorylation of p44 (ERK1) and p42 (ERK2). Data are expressed as **(a) **immunoblots with levels of phosphorylated mitogen-activated protein kinase (MAPK) in the top band and total loading of protein below and as **(b) **mean percentage of the unstimulated basal levels of MAPK phosphorylation ± standard error of the mean (n = 3 synovia). Comparison between drug treatment groups was carried out using one-way analysis of variance. **P < 0.05 versus HU210. ERK, extracellular signal-regulated kinase.

Levels of phosphorylated and total ERK1, ERK2 (p44 and p42, respectively), and p38 MAPK were measured in fibroblast-like cells from OA and RA patients, derived from the synovial tissue, by Western blotting. Given the comparable levels of expression of CB_1 _and CB_2 _receptor protein in OA and RA samples, we combined RA and OA cells to maximise cell yield for these pharmacological experiments. The non-selective cannabinoid receptor agonist HU210 (1 μM) produced a time-dependent phosphorylation of ERK1, ERK2, and p38 MAPK, indicating an increase in ERK and p38 activity which peaked at 10 minutes after stimulation (Figure 6). Levels of total ERK1, ERK2, and p38 were unaffected by HU210 (data not shown). Pre-treatment of fibroblast-like cells with PTX, which ADP-ribosylates and inactivates Gα_i/o_, decreased HU210-induced phosphorylation. The effect of PTX was significant for p38 activation after a 10-minute stimulation with HU210 (*P *< 0.05); although PTX showed a tendency to decrease HU210-induced activation of ERK1 and ERK2, significance was not reached. These data suggest a role for a Gα_i/o_-coupled receptor mediating the effects of HU210 on ERK1, ERK2, and p38 activation (Figure 6). To further investigate the role of the cannabinoid receptors in mediating the effects of HU210 on phosphorylation of ERK1, ERK2, and p38 MAPK, the potential ability of the CB_1 _and CB_2 _receptor antagonists SR141716A and SR144528 to block the effects of HU210 (0.1 μM) was studied. The CB_1 _receptor antagonist SR141716A (1 μM) significantly attenuated HU210 (0.1 μM)-induced phosphorylation of ERK1 and ERK2 in fibroblast-like cells (Figure 7). Although the CB_2 _receptor antagonist SR144528 (1 μM) tended to attenuate HU210 (0.1 μM)-induced phosphorylation of ERK1 and ERK2 in fibroblast-like cells, significance was not reached (Figure 7). Levels of total ERK1 and ERK2 were unaffected by the drug treatments (Figure 7a). HU210-induced phosphorylation of p38 MAPK was not significantly attenuated by the CB_1 _or CB_2 _receptor antagonist (data not shown). Overall, these pharmacological studies provide strong support for functionally coupled cannabinoid receptors in the fibroblast-like cells derived from synovia from OA and RA patients.

## Discussion

The novel finding of the present study is the identification of the key components of the cannabinoid receptor system in the knee synovia of patients with end-stage OA and RA. We have demonstrated, for the first time, the presence of cannabinoid CB_1 _and CB_2 _receptor message and protein. The functional relevance of the presence of these receptors has been confirmed by pharmacological studies demonstrating cannabinoid agonist-induced phosphorylation of the downstream signalling targets (ERK1 and ERK2) in fibroblast-like cells derived from OA and RA patients. The endocannabinoids, plus associated entourage compounds and FAAH activity, were present in the synovia of both OA and RA patients. In addition, we have demonstrated that AEA and 2-AG are also present in the synovial fluid of OA and RA patients but are not detectable in synovial fluid taken from volunteers with no joint symptoms. Our data provide evidence for a functional endocannabinoid receptor system in OA and RA patients.

All synovia used in the present study were collected from RA and OA patients with end-stage disease undergoing TKA for severe pain. Histological analysis verified that the synovia were not normal. Both the OA and RA synovia exhibited either moderate or severe inflammation. Moderate or severe synovitis was classified as the intima layer being more than four cells deep, plus dense cellularity of subintimal tissue due to increased numbers of fibroblastic cells and inflammatory cells, including lymphoid aggregates [3,44,45]. In general, the number of lymphoid aggregates and cell depth of the synovial intima are greater, or more extensive, in RA than OA synovium [46,47]. All of the RA and OA patients whose samples were used in this study exhibited severe disease and there were no significant differences between levels of cytokines in RA and OA samples studied. Levels of IL-6, however, were significantly higher in OA and RA samples compared with volunteers with no joint symptoms. IL-6 is an important driver of inflammation in RA [48] and all of the synovia, whether RA or OA, were inflamed in our study. IL-6 is also implicated in the induction of osteoclast differentiation and bone resorption [49], and all of our patients had bone-on-bone changes somewhere within their arthritic knees, reflecting the severity of end-stage disease requiring joint replacement surgery. Reported levels of IL-6 [50,51] and IL-8 [51,52] are in keeping with earlier reports in OA and RA.

Although it may be expected that levels of pro-inflammatory cytokines would be higher in RA samples, all of the patients had taken anti-arthritis medicines, which could affect levels of cytokines and possibly endocannabinoids. As a result of the different requirements for sample preparation and the amount of synovia available, not all of the synovia could be used for all of the experimental studies. Given the wide range of cytokine levels present in OA and RA samples, we have studied the cannabinoid receptor system in groups of OA and RA samples which represent a cross-section of the population in terms of levels of cytokines, ensuring that our data were not subject to bias. Due to difficulties in recruiting male RA subjects, only one was included in the study, but similarities between the extent of disease in the male and female subjects and the lack of significant difference between cytokine levels in RA and OA samples suggest that this should not confound our data.

Here, we report the presence of both the CB_1 _and CB_2 _receptors in the synovia of patients with end-stage OA and RA, suggesting that this system may play a role in these pathological conditions. Our pharmacological study demonstrating that the potent cannabinoid agonist HU210 phosphorylates ERK1 and ERK2 in fibroblast-like synovial cells in a PTX-dependent manner via the CB_1 _receptor lends further support to a functional role of this receptor system in OA and RA synovia. Although there was a trend toward an attenuation of the effects of HU210 by the CB_2 _receptor antagonist, significance was not reached. Pre-clinical studies have demonstrated that activation of CB_1 _receptors, both on peripheral nerves and at spinal and supraspinal sites, produces analgesic effects in models of acute and inflammatory pain [53-55]. By contrast, CB_2 _receptors are associated predominantly with immune cells (see Introduction). Although, in the present study, the cellular location of the cannabinoid receptors has not been identified, the demonstration that cannabinoid receptors are coupled to the MAPK signalling pathway in fibroblast-like cells prepared from OA and RA synovia indicates that these cells are a likely location for the cannabinoid receptors identified.

The two main endocannabinoids, AEA and 2-AG, were present in the synovia of OA and RA patients at levels in keeping with those previously reported in other biological tissues. The fatty acid amides PEA and OEA [56] were also detected in both OA and RA synovia. PEA is of particular interest since it has anti-inflammatory activity [57] via nuclear PPAR-α (peroxisome proliferator activating receptor-α) activation and possibly endocannabinoid entourage effects. Unfortunately, it was not possible to obtain non-diseased synovia and, therefore, a comparison of levels of ECs in normal synovium with OA and RA samples was not possible. However, we were able to compare levels of endocannabinoids in the synovial fluid, which contains immune cells that are capable of releasing endocannabinoids [9], of OA and RA patients compared with normal volunteers. AEA and 2-AG were present in the synovial fluid of OA and RA patients, but not in normal controls. Levels of 2-AG were significantly lower in the RA group compared with the OA group. Levels of PEA were significantly lower in the synovial fluid of OA and RA patients compared with that of non-inflamed normal volunteers. Since PEA has a well-described anti-inflammatory role, the reported lower levels of PEA in the synovial fluid of OA and RA patients may contribute to the disease process associated with these conditions. Given that AEA, PEA, and OEA are all substrates for FAAH, the opposing impact of OA/RA on levels of these compounds suggests that these changes are not due merely to alterations in FAAH-mediated metabolism and argues against an important contribution of the entourage effect. Levels of FAAH activity were comparable in OA and RA samples. Although activity of FAAH in the synovium was low relative to the liver, it was comparable to levels previously described in rat hindpaw [34]. FAAH activity was undetectable in the presence of pharmacological blockade of FAAH by the well-characterised inhibitor of URB597 [58], indicating the functional relevance of this activity. On the basis of our FAAH activity data, changes in rates of synthesis or release of AEA and 2-AG, versus PEA and OEA, in OA and RA patients compared with non-inflamed normal volunteers are more likely to account for our data.

In some cases, the relative levels of endocannabinoids and related fatty acid amides in the synovial fluid did not mirror levels in the synovia of OA and RA patients. Levels of 2-AG in the synovial fluid of RA patients were significantly lower than levels in OA patients, whereas there were no differences in levels of 2-AG in the synovia of OA and RA patients. In addition, levels of PEA were non-significantly higher in RA synovium compared with OA synovium, but levels of PEA were similar in the synovial fluid of OA and RA patients. Thus, levels in the synovial fluid do not simply reflect the level of synthesis/release and catabolism of endocannabinoids and entourage compounds in the synovium. The source of the endocannabinoids present in the synovium and synovial fluid is an important consideration. Endocannabinoids are synthesised by numerous different cell types, including immune cells such as T cells and macrophages, which are the major immune cells present in OA and RA [47,59,60]. Since endothelial cells can synthesise AEA [61] and 2-AG [62], another possible source of endocannabinoids in the synovium is the vasculature. The role of vascular elements in the progression of arthritic disease is important, particularly since neovascularisation is one of the early changes in the synovium and it is thought that bone and cartilage destruction is closely linked to angiogenesis [63] and cannabinoids inhibit angiogenesis in tumours [37,64].

## Conclusion

In summary, cannabinoid CB_1 _and CB_2 _receptor protein and RNA and the endocannabinoids AEA and 2-AG are present in the synovia of patients with end-stage OA and RA. The presence of increased levels of AEA and 2-AG in the synovial fluid of OA and RA patients, compared with non-inflamed normal volunteers, suggests a greater functional role of the endocannabinoid receptor system in these patients. Importantly, levels of the anti-inflammatory substance PEA were higher in the synovial fluid of normal volunteers compared with OA and RA patients and, therefore, the loss of PEA may contribute to arthritic disease. Our data predict that the cannabinoid receptor system may be an important therapeutic target for the treatment of pain and inflammation associated with these conditions.

## Abbreviations

2-AG = 2-arachidonyl glycerol; AEA = anandamide; CB_1 _= cannabinoid receptor 1; CB_2 _= cannabinoid receptor 2; COX = cyclooxygenase; EC = endocannabinoid; EGTA = ethylene glycol tetraacetic acid; ERK = extracellular signal-regulated kinase; FAAH = fatty acid amide hydrolase; IL = interleukin; MAPK = mitogen-activated protein kinase; NSAID = non-steroidal anti-inflammatory drug; OA = osteoarthritis; OEA = oleoyl ethanolamide; PCR = polymerase chain reaction; PEA = palmitoyl ethanolamide; PTX = pertussis toxin; RA = rheumatoid arthritis; TKA = total knee arthroplasty; TNF = tumour necrosis factor; URB597 = 3'-(aminocarbonyl) [1,1'-biphenyl]-3-yl)-cyclohexylcarbamate.

## Competing interests

The authors declare that they have no competing interests.

## Authors' contributions

DR performed endocannabinoid measurements and statistical analyses and contributed to the writing of the manuscript. RGP prepared tissue for cytokine analysis, performed histology and statistical analyses, and contributed to the writing of the manuscript. DR and RGP are joint first authors. NK performed the reverse transcription-polymerase chain reaction, FAAH, and MAPK assays and associated statistical analyses. LL performed CB_1_/CB_2 _Western blotting and statistical analyses. MJG contributed to the FAAH assay. DAB designed research and contributed to endocannabinoid measurements. DAK designed research and contributed to the writing of the manuscript. BES designed research, performed TKA and histology scoring, and contributed to the writing of the manuscript. AJR contributed to the writing of the manuscript. VC designed research and significantly contributed to the writing of the manuscript. All authors read and approved the final manuscript.
